# Patterns of human social contact and mask wearing in high-risk groups in China

**DOI:** 10.1186/s40249-022-00988-8

**Published:** 2022-06-18

**Authors:** Bo Zheng, Wenlong Zhu, Jinhua Pan, Weibing Wang

**Affiliations:** 1 Department of Epidemiology, School of Public Health, Fudan University, Shanghai, 200032 China; 2 Shanghai Institute of Infectious Disease and Biosecurity, School of Public Health, Fudan University, 138 Yi Xue Yuan Road, Shanghai, 200032 China; 3 Key Lab of Public Health Safety of the Ministry of Education, Fudan University, Shanghai, 200032 China

**Keywords:** COVID-19, Human–human contact pattern, High-risk population

## Abstract

**Background:**

The pandemic of coronavirus disease 2019 (COVID-19) has changed human behavior in areas such as contact patterns and mask-wearing frequency. Exploring human–human contact patterns and mask-wearing habits in high-risk groups is an essential step in fully understanding the transmission of respiratory infection-based diseases. This study had aims to quantify local human–human (H–H) contacts in high-risk groups in representative provinces of China and to explore the occupation-specific assortativity and heterogeneity of social contacts.

**Methods:**

Delivery workers, medical workers, preschoolers, and students from Qinghai, Shanghai, and Zhejiang were recruited to complete an online questionnaire that queried general information, logged contacts, and assessed the willingness to wear a mask in different settings. The “group contact” was defined as contact with a group at least 20 individuals. The numbers of contacts across different characteristics were assessed and age-specific contact matrices were established. A generalized additive mixed model was used to analyze the associations between the number of individual contacts and several characteristics. The factors influencing the frequency of mask wearing were evaluated with a logistic regression model.

**Results:**

A total of 611,287 contacts were reported by 15,635 participants. The frequency of daily individual contacts averaged 3.14 (95% confidence interval: 3.13–3.15) people per day, while that of group contacts was 37.90 (95% *CI*: 37.20–38.70). Skin-to-skin contact and long-duration contact were more likely to occur at home or among family members. Contact matrices of students were the most assortative (all contacts *q*-index = 0.899, 95% *CI*: 0.894–0.904). Participants with larger household sizes reported having more contacts. Higher household income per capita was significantly associated with a greater number of contacts among preschoolers (*P*_50,000–99,999_ = 0.033) and students (*P*_10,000–29,999_ = 0.017). In each of the public places, the frequency of mask wearing was highest for delivery workers. For preschoolers and students with more contacts, the proportion of those who reported always wearing masks was lower (*P* < 0.05) in schools/workplaces and public transportation than preschoolers and students with fewer contacts.

**Conclusions:**

Contact screening efforts should be concentrated in the home, school, and workplace after an outbreak of an epidemic, as more than 75% of all contacts, on average, will be found in such places. Efforts should be made to improve the mask-wearing rate and age-specific health promotion measures aimed at reducing transmission for the younger demographic. Age-stratified and occupation-specific social contact research in high-risk groups could help inform policy-making decisions during the post-relaxation period of the COVID-19 pandemic.

**Graphical Abstract:**

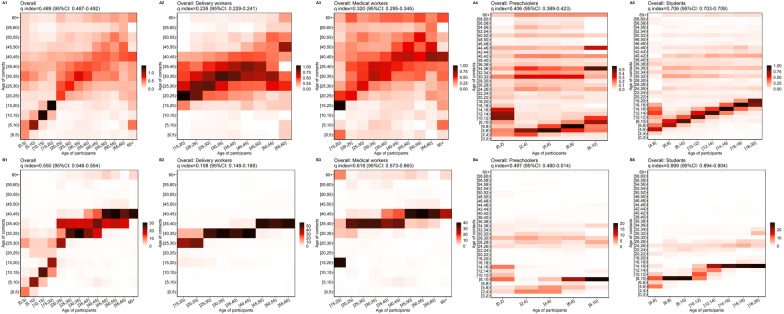

**Supplementary Information:**

The online version contains supplementary material available at 10.1186/s40249-022-00988-8.

## Background

Respiratory-borne diseases, such as mumps, influenza, and chickenpox [1–3] and relevant emerging pathogens with established human–human transmission [e.g., coronavirus disease 2019 (COVID-19)] can spread through the exchange of respiratory droplets between people in close physical proximity to one another [4, 5]. Since this transmission pattern is relevant to self-reported contacts, the human spread or even outbreak of these diseases is likely to be driven by patterns of human encounters [6]. It is important to quantify these interactions, especially in light of how different age groups mix; these data can be used to increase the effectiveness of disease-targeting interventions, such as vaccination, contact tracing, and social distancing [7], and generate mathematical models that can predict the course of an epidemic and the effectiveness of interventions [8]. For example, the famous POLYMOD study investigated the social contact patterns in eight European countries, combined the findings with serological data, and found that intimate contact can explain the transmission of varicella and parvovirus B19 infection [2]. Dodd et al*.* used social contact pattern data to enumerate “close” (shared conversation) and “casual” (shared indoor space) social contacts in 16 Zambian communities and eight South African communities to model the incidence of *Mycobacterium tuberculosis* infection among adults [9]. Data on human interaction patterns can therefore help researchers clarify risk factors for transmission and design interventions for controlling epidemics.

However, most of the relevant researches were conducted before the outbreak of COVID-19 and the ensuing implementation of control measures. These measures have reshaped the behavior patterns of Chinese society [10], especially in terms of mask wearing and social distancing. In addition, few (if any) of the existing surveys focused on high-risk people, such as delivery workers, medical workers (who interact in large groups), or school-aged children (who generally have low levels of prior immunity [11]). Finally, the rapid economic development, high urbanization, and frequent human interactions in China [12] mean that this country plays an important role in global pandemics of respiratory-transmitted diseases, such as influenza [13]. Thus, efforts to identify new accurate parameters for contact patterns in high-risk groups after the COVID-19 outbreak in China will be critical for improving the accuracy of mathematical models in predicting the spread of infections and assessing preventive measures [14].

Here, we selected districts/counties of Shanghai, Zhejiang Province, and Qinghai Province as our study sites. A diary-based survey was employed to survey social contacts at these sites between December 2020 and March 2021. Our contact study not only focused high-risk population, but also investigated at the national level after the outbreak of COVID-19. This study had three aims: (1) to quantify local human–human (H–H) contacts in high-risk groups in representative provinces of China, (2) to explore the occupation-specific assortativity and heterogeneity of social contacts, and (3) to assess the behavioral pattern of mask wearing under the existing COVID-19 prevention and control measures.

## Methods

### Study sites and sampling

Our survey was carried out between December 2020 and March 2021 in three provincial-level administrative divisions of China (Shanghai, Zhejiang, and Qinghai), which were chosen for each having different population densities and socioeconomic factors, allowing for greater representativeness of the overall data. The Minhang and Songjiang Districts in Shanghai City, Huzhou City in Zhejiang Province, and Haidong City and Haixizhou City in Qinghai Province were purposively selected as survey sites. From these sites, sufficient districts/counties were sampled using multi-stage stratified sampling. Lists of hospitals, schools (kindergartens, primary, junior, and senior schools) and delivery companies were obtained from each district/county. Lists were randomized, and recruitment of eligible individuals was attempted in sequence from this list with the help of local district/county workers. The high-risk populations targeted in the study were delivery workers, medical workers, preschoolers, and students. Preschoolers, mostly aged 0–5 years, are children who have not entered elementary school and students includes elementary, junior, and senior high school students, who are generally aged 6–19. We did not set any maximum target sample size for each specific institution. According to our calculations, the sample size of this cross-sectional study should have been at least 9811 participants, of whom 20% might fail to complete the survey. Thus, we needed to recruit at least 818 participants from each group per site. An online questionnaire was used to collect the relevant information, and all obtained questionnaires were subject to quality audit by the investigator.

For delivery workers, we sampled several delivery companies per site and established WeChat groups for each of them. WeChat groups are composed of many individual users and are widely used as a daily social communication platform for people in China. Assisted by delivery company managers, we tried to recruit all the delivery workers of selected companies to enter our WeChat groups. A trained investigator joined each WeChat group, introduced the purpose of the study, and provided information on filling out the questionnaire. The delivery workers who consented to participate in the survey were asked to fill in a web-based questionnaire. Medical workers from one, two, or three hospitals in each survey site were selected as participants and asked to complete the survey. Preschoolers were recruited from one or two kindergartens of each site. At each of Shanghai, Zhejiang, and Qinghai, primary, junior, and senior schools (one each) were selected, and students from one class of each grade in the school were included. Parents of preschoolers and primary school students were asked to recall their child’s contacts and complete the questionnaire. Junior and senior school students were asked to self-complete the questionnaire after their parents’ informed consent was obtained. For all participants included in the study, an informed consent form was obtained from the participant or their parents (preschoolers and primary school students). All included participants had lived in the district/county for at least 1 month prior to being enrolled in the study.

### Survey contents and methods

The questionnaire consisted of three sections: general information, contact frequency, and willingness to wear a mask in different settings (see Additional file 1). General information comprised the respondent’s demographics, including their age, sex, income, duration of local living, and household size. Referring to POLYMOD [6], contact was defined as: (1) a two-way conversation with three or more words in the physical presence of another person (conversational touch), or (2) skin-to-skin contact (such as a handshake, hug, kiss, or contact sport). In the contact diary, for “individual contacts” (those occurring with up to 19 persons), participants were asked to list each person with whom they had contact during a day and give some details about each contacted individual, including their age (or age range, which was replaced by the mid-point of the range for the purposes of analysis), sex, relationship to respondent (relative, colleague/classmate, friend, teacher, or other), contact type (physical contact or not), setting of the encounter (home, school, office, transportation, or other), duration (less than 5 min, 5–15 min, 15–60 min, 1–4 h, more than 4 h) and frequency (almost every day, once or twice per week, once or twice per month, less than once per month, first meeting). If a participant had contact with the same person several times in a given day, it was recorded as one contact and the total duration of all interactions was used. As this item-by-item approach is inappropriate in recording contacts with multiple people over a period of time, we defined “group contact” as contact with a group at least 20 individuals, i.e., due to occupation or participation in some activity. For group contacts, participants reported the number of age-specific group contacts per day. Regarding mask wearing, for each of the listed places (school/workplace, public transportation, training institution, outdoor public space, non-enclosed indoor public space, enclosed indoor public space, medical place) visited in the prior month, participants were asked to report their frequency of mask wearing (never, rarely, occasionally, often, every time) at the site.

This survey was designed to be fully anonymous and the names of participants were not recorded at any point. The study received ethical approval from the School of Public Health, Fudan University.

### Statistical analysis

The distribution of contact numbers was assessed for each region, occupation, sex, age group, education level, household income per capita, and household size. For individual contacts, the proportions of contact duration, setting, relationship, and frequency were plotted. Proportions of different contact settings and relationships were also stratified by contact duration and frequency.

We established different age classes among the different occupations and regions to build our age-specific H–H contact matrices, with the goal of estimating the age-specific individual/overall contact number per participant per day. Participants did not report the exact age of each member of a group contact, so we modeled the age distribution for individual contacts using the Gaussian kernel function, stratified by the age groups of participants in each occupation (Additional file 1: Fig. S1). Then we drew the age of group contacts randomly from the model, with reference to the age group of the participants and the group contacts. We repeated the sampling process 200 times to estimate uncertainty. We used *q*-indices, which represented departures from proportionate mixing and ranged from zero (proportionate) to one (fully assortative), and bootstrapped 95% confidence intervals to assess the degree of age assortativity [15].

A Generalized Additive Mixed Model (GAMM) with a negative binomial distribution was used to analyze the association between the number of total contacts (individual and group contact) and the selected variables (sex, household size, household income per capita, weekdays or weekend days, and region) in the different occupation groups. We fitted thin plate regression splines to explore potential nonlinear relationships between continuous participant age and the contact number.

Chi-squared tests were used to compare the distribution of mask wearing between regions and settings among the four occupations. We divided mask wearing into two levels (wearing a mask every time or not at all) and used univariate logistic regression to analyze factors that might affect mask wearing. Independent variables (occupation, contact group) were included in our multinomial logistic regression model.

Data analyses were performed using the R.4.1.1 software (Foundation for Statistical Computing, Vienna, Austria. ISBN 3-900051-07-0, URL http://www.R-project.org) with the *mgcv* and *socialmixr* packages. All figures were plotted using the R package, *ggplot2.* Differences were considered statistically significant at *P* < 0.05.

## Results

### Demographic characteristics of participants

We collected data from 15,635 participants (Table 1); 51.6% were male and 70.4% were under 20 years of age. Of the participants, 9.5%, 12.2%, 29.5%, and 48.8% were delivery workers, medical workers, preschoolers, and students, respectively. Household income per capita was above CNY 50,000 for 35.5% of the participants. Most (47.6%) of the participants were members of households having three or four members. Characteristics of participants stratified by occupation and province are presented in Additional file 2: Table S1.

**Table 1:** Number of contacts by demographic characteristics, occupations, and locations

Characteristics		Number of participants *n* (%)	Individual contacts Mean (95% *CI*)	Group contacts Mean (95% *CI*)	Total contacts Mean (95% *CI*)
Overall		15,635	3.14 (3.13–3.15)	37.90 (37.20–38.70)	41.10 (40.37–41.83)
Sex	Male	8066 (51.6)	3.14 (3.13–3.15)	39.72 (38.63–40.81)	42.85 (41.76–43.94)
	Female	7569 (48.4)	3.14 (3.13–3.15)	36.09 (35.13–37.06)	39.23 (38.27–40.20)
Age	[0, 5]	2844 (18.2)	3.20 (3.17–3.23)	53.39 (49.70–57.08)	56.59 (52.90–60.28)
	[5, 10]	4314 (27.6)	3.17 (3.14–3.19)	54.78 (50.83–58.73)	57.95 (54.00–61.90)
	[10, 15]	3842 (24.6)	3.18 (3.14–3.22)	55.21 (49.47–60.95)	58.39 (52.65–64.13)
	[15, 20]	1269 (8.1)	3.13 (3.11–3.15)	39.27 (36.99–41.54)	42.40 (40.12–44.67)
	[20, 30]	1356 (8.7)	3.20 (3.13–3.28)	48.45 (39.23–57.67)	51.65 (42.42–60.88)
	[30, 40]	1240 (7.9)	3.10 (3.09–3.11)	33.06 (32.11–34.02)	36.16 (35.20–37.12)
	[40, 50]	589 (3.8)	3.15 (3.13–3.16)	25.16 (24.19–26.13)	28.30 (27.33–29.27)
	50 +	181 (1.2)	3.14 (3.12–3.15)	38.50 (37.10–39.91)	41.64 (40.23–43.05)
Occupation	Delivery workers	1491 (9.5)	3.13 (3.11–3.15)	62.82 (58.76–66.87)	65.95 (61.89–70.01)
	Medical workers	1904 (12.2)	3.22 (3.20–3.25)	47.19 (44.50–49.87)	50.41 (47.73–53.10)
	Preschoolers	4616 (29.5)	3.13 (3.12–3.15)	25.74 (24.99–26.50)	28.88 (28.12–29.63)
	Students	7624 (48.8)	3.12 (3.11–3.13)	38.20 (37.28–39.12)	41.32 (40.40–42.24)
Household income per capita, CNY	< 10,000	4744 (30.3)	3.15 (3.13–3.16)	41.20 (39.35–43.04)	44.34 (42.50–46.18)
	10,000–29,999	2910 (18.6)	3.13 (3.12–3.15)	38.75 (37.00–40.50)	41.88 (40.13–43.64)
	30,000–49,999	2464 (15.8)	3.12 (3.11–3.13)	36.96 (35.58–38.34)	40.08 (38.70–41.46)
	50,000–99,999	2907 (18.6)	3.14 (3.13–3.16)	37.32 (35.69–38.94)	40.46 (38.83–42.09)
	≥ 100,000	2610 (16.7)	3.16 (3.14–3.18)	36.16 (34.54–37.79)	39.32 (37.70–40.95)
Household size	1–2	5695 (36.4)	3.14 (3.12–3.15)	40.77 (39.40–42.14)	43.91 (42.54–45.28)
	3–4	7440 (47.6)	3.13 (3.12–3.14)	36.30 (35.34–37.27)	39.43 (38.47–40.40)
	≥ 5	2500 (16.0)	3.16 (3.14–3.18)	36.50 (34.81–38.20)	39.66 (37.96–41.36)
Province	Qinghai	8437 (54.0)	3.14 (3.12–3.15)	44.70 (42.48–46.93)	47.84 (45.62–50.07)
	Shanghai	2978 (19.0)	3.12 (3.11–3.13)	35.33 (34.48–36.19)	38.46 (37.60–39.31)
	Zhejiang	4220 (27.0)	3.17 (3.15–3.18)	38.46 (37.07–39.85)	41.62 (40.24–43.01)

*N is the number of participants who provided complete contact data. *CI* confidence interval, *CNY* Chinese Yuan

### Characteristics of human–human contact patterns

**Fig. 1:**
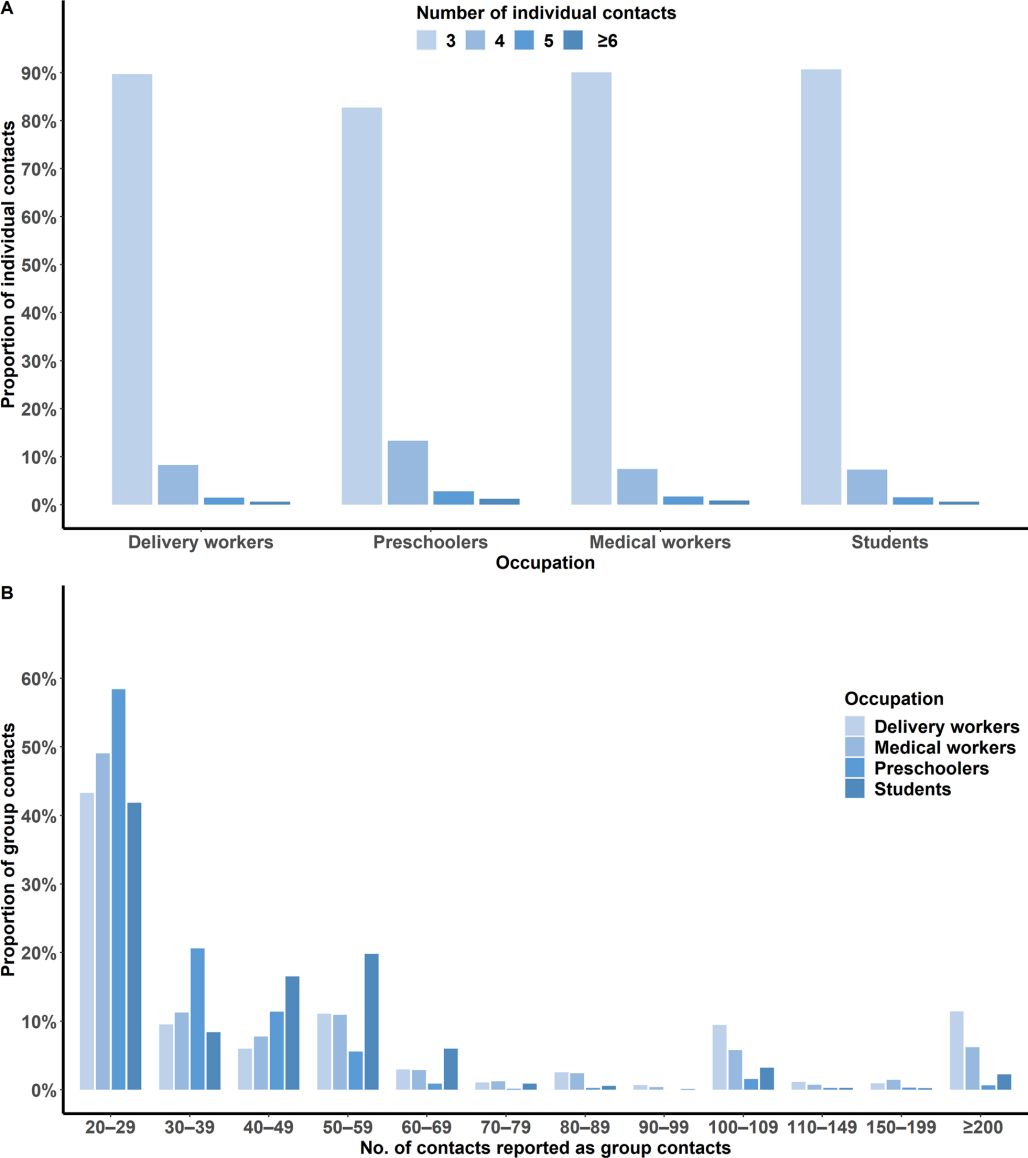
Distribution of individual contacts (**A**) and group contacts (**B**) stratified by occupation

A total of 611,287 contacts were reported by 15,635 participants in Qinghai, Shanghai, and Zhejiang; of them, 92.0% were group contacts. There was little difference in the probability of the number of individual contacts reported in terms of the participant’s occupation. The number of individual contacts was around 3.14 individuals per day (95% *CI*: 3.13–3.15) (Table 1). With respect to the four occupational groups, 90.6% of students had individual contacts with 3 people per day, compared to 90.0% of preschoolers, 89.7% of delivery workers, and 82.7% of medical workers (Fig. 1A1). Males reported more group contacts, and participants under 15 years old reported more than 50 group contacts per day. Delivery workers reported most group contacts [62.82 (95% *CI*: 58.76–66.87)], while preschoolers reported the fewest [25.74 (95% *CI*: 24.99–26.50)]. Participants whose households had one or two members reported more group contacts. Participants from Shanghai reported the fewest group contacts among the sites (Table 1). The distribution of the number of group contacts was right-skewed among the different occupations. Among the occupation groups, more than 40.0% of participants reported 20–29 group contacts; moreover, 22.9% of delivery workers and 14.1% of medical workers reported more than 100 group contacts, compared with 2.8% of preschoolers and 5.9% of primary and secondary school students (Fig. 1A2). The average daily number of contacts per participant differed by (when reported) sex, age, education level, household income per capita, and household size among the four occupations in the three provinces (Additional file 2: Table S1). Among delivery workers, males reported more contacts than females (*P*_Qinghai_ = 0.046, *P*_Shanghai_ < 0.001, *P*_Zhejiang_ < 0.001). Among medical workers, preschoolers, and students, the number of contacts did not significantly differ between males and females. Younger delivery workers aged 18–34 had contact with more people than their older counterparts, and medical workers aged 18–24 and 35–44 had contact with more people than their older counterparts. Among preschoolers and students, the older the respondent, the more contacts were reported. In the four occupation groups, the trends for the contact number and household size differed across the three sites (Additional file 1: Table S1).

**Fig. 2:**
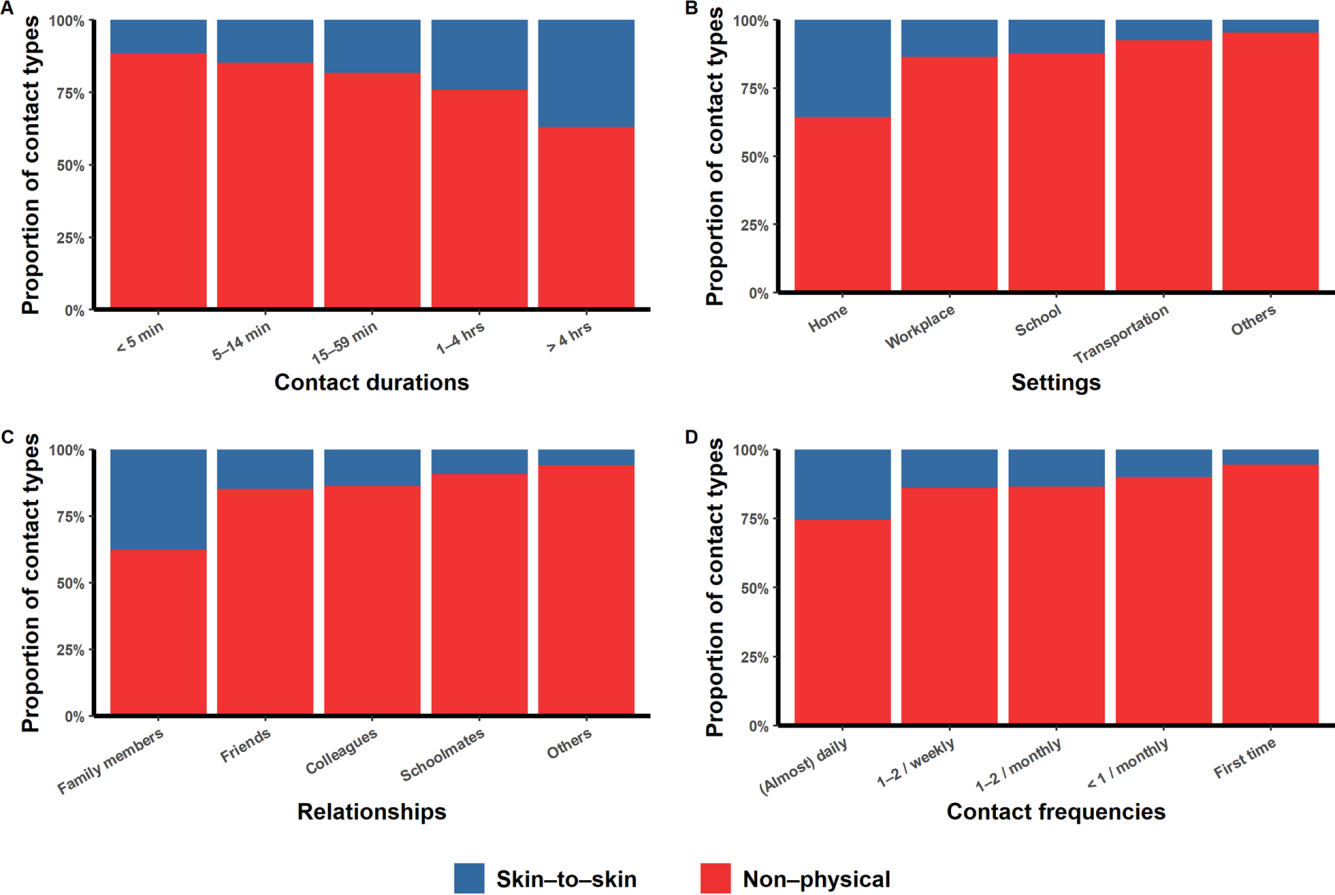
The proportions of physical contacts for different contact durations (**A**), settings (**B**), relationships (**C**), and frequencies (**D**)

**Fig. 3:**
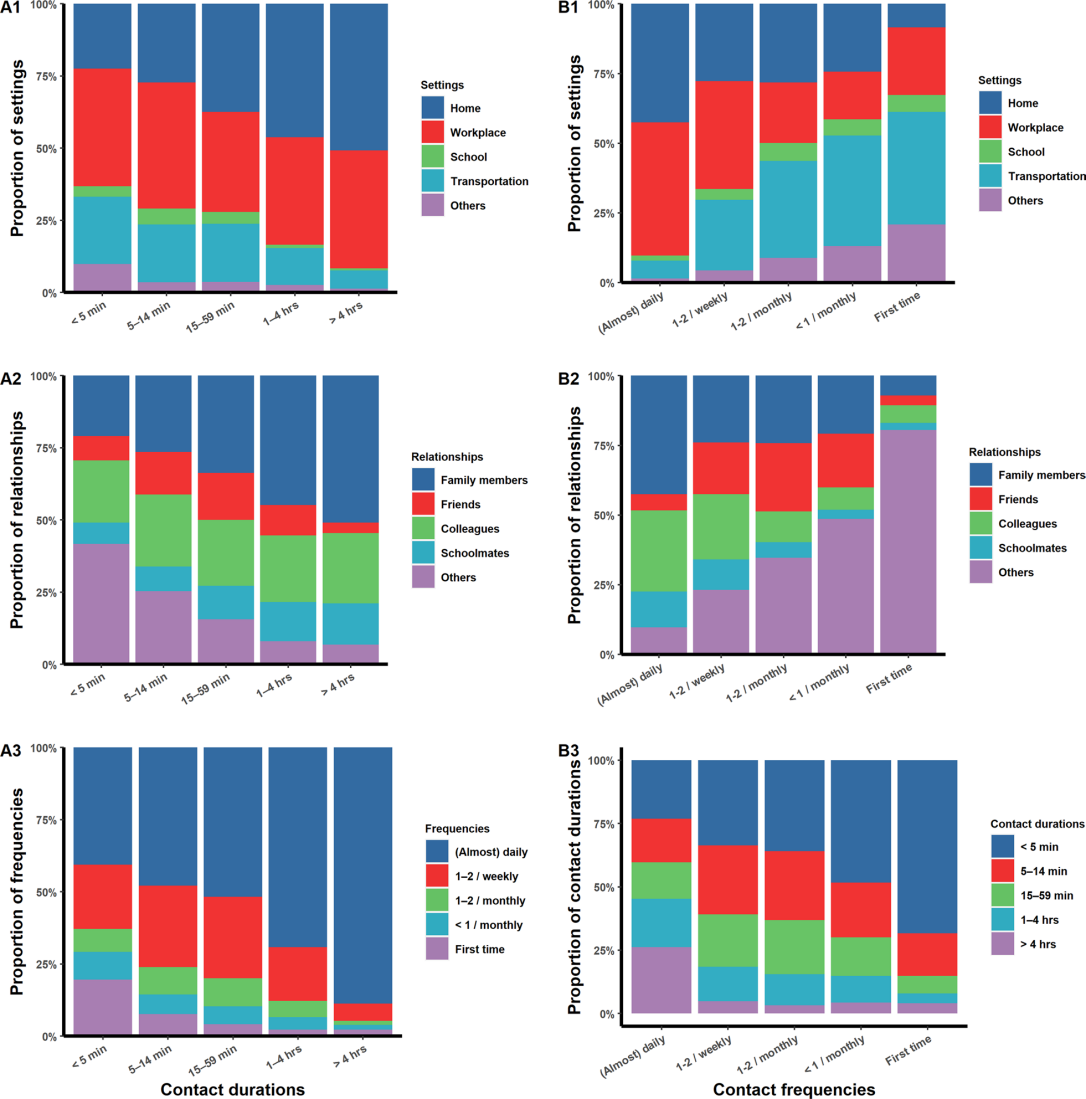
Distribution of individual contact patterns. **A1**–**A3** The proportions of contact durations according to individual contact settings (**A1**), relationships (**A2**) or frequencies (**A3**). **B1**–**B3** The proportions of contact frequencies according to individual contact settings (**B1**), relationships (**B2**) or contact durations (**B3**)

For all participants, the proportion of direct contact increased with the contact duration (Fig. 2A), and the proportion of skin-to-skin contact was higher when we considered contact occurring at home or between family members (Fig. 2B, C). Fewer skin-to-skin contacts occurred for first-time contacts (Fig. 2D). The proportions of physical contacts stratified by occupation or province showed the abovementioned patterns (Additional file 1: Fig. S2 − S5). As the contact duration increased, so did the proportion of contacts happening at home and among family members (Fig. 3A1, A2). For most (88.7%) of the participants with contact durations > 4 h, the involved contacts were daily contacts (Fig. 3A3). Of the daily contacts, 90.3% happened at home or in the workplace (Fig. 3B1). Less frequent contacts were less likely to be with family members or colleagues (Fig. 3B2). Contact duration also declined as the contact frequency decreased (Fig. 3B3).

### Human–human contact matrix and assortativity of contacts

**Fig. 4:**
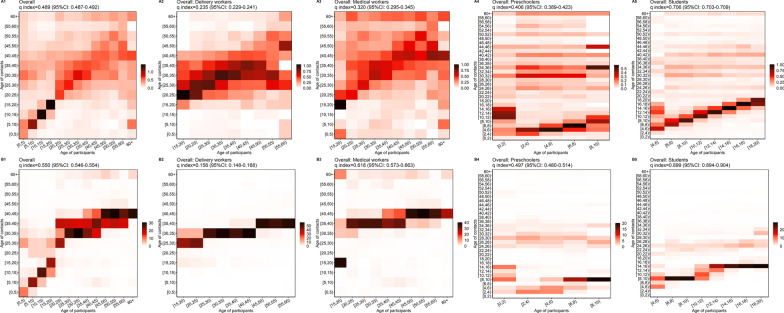
Contact matrices by age. Each cell of the matrix represents the mean number of contacts that an individual in a given age group has with other individuals, stratified by age group. The color intensity represents the number of individual contacts (**A1**–**A5**) or all contacts (**B1**–**B5**). To construct the matrix, we performed bootstrap sampling with replacement of survey participants weighted by the age distributions of the actual populations of Shanghai, Zhejiang, and Qinghai. Each cell of the matrix represents an average over 100 bootstrapped realizations

The overall *q*-indexes for individual contacts and all contacts were 0.489 (95% *CI*: 0.487–0.492) and 0.550 (95% *CI*: 0.546–0.554), respectively. There was a diagonal element in the overall individual contact matrix (Fig. 4A1), indicating that participants in different age groups trended to mix assortatively by age. The diagonal element was most pronounced in those aged 5–20 years, and least pronounced in those aged 45 years and above. For the general contact matrix (Fig. 4B1), the diagonal element was most pronounced in those aged 0–20 years. Participants aged 20 years and above mainly had contact with individuals aged 25–45 years. For individual contacts and all contacts (Fig. 4A5, B5), the contact matrices of students were the most assortative (individual contacts *q*-index = 0.706, 95% *CI*: 0.703–0.709; all contacts *q*-index = 0.899, 95% *CI*: 0.894–0.904). The assortativity was weakest among delivery workers (Fig. 4A2, B2). Delivery workers had fewer contacts with individuals aged 0–15 years and more contacts with individuals aged 25–40 years. Preschoolers and students tended to have contact with the same-age individuals and those aged 24–40 years. For medical workers, the individual contacts (Fig. 4A3) revealed a diagonal starting around 15–50 years old for both contacts and participants. The patterns described above were also observed in the contact matrices stratified by province and occupation (Additional file 1: Fig. S6, S7). Among the three provinces, Qinghai showed more assortativity (individual contacts *q*-index = 0.543, 95% *CI*: 0.543–0.548; all contacts *q*-index = 0.641, 95% *CI*: 0.632–0.649) than the other two provinces.

### Factors associated with contact frequency

**Fig. 5:**
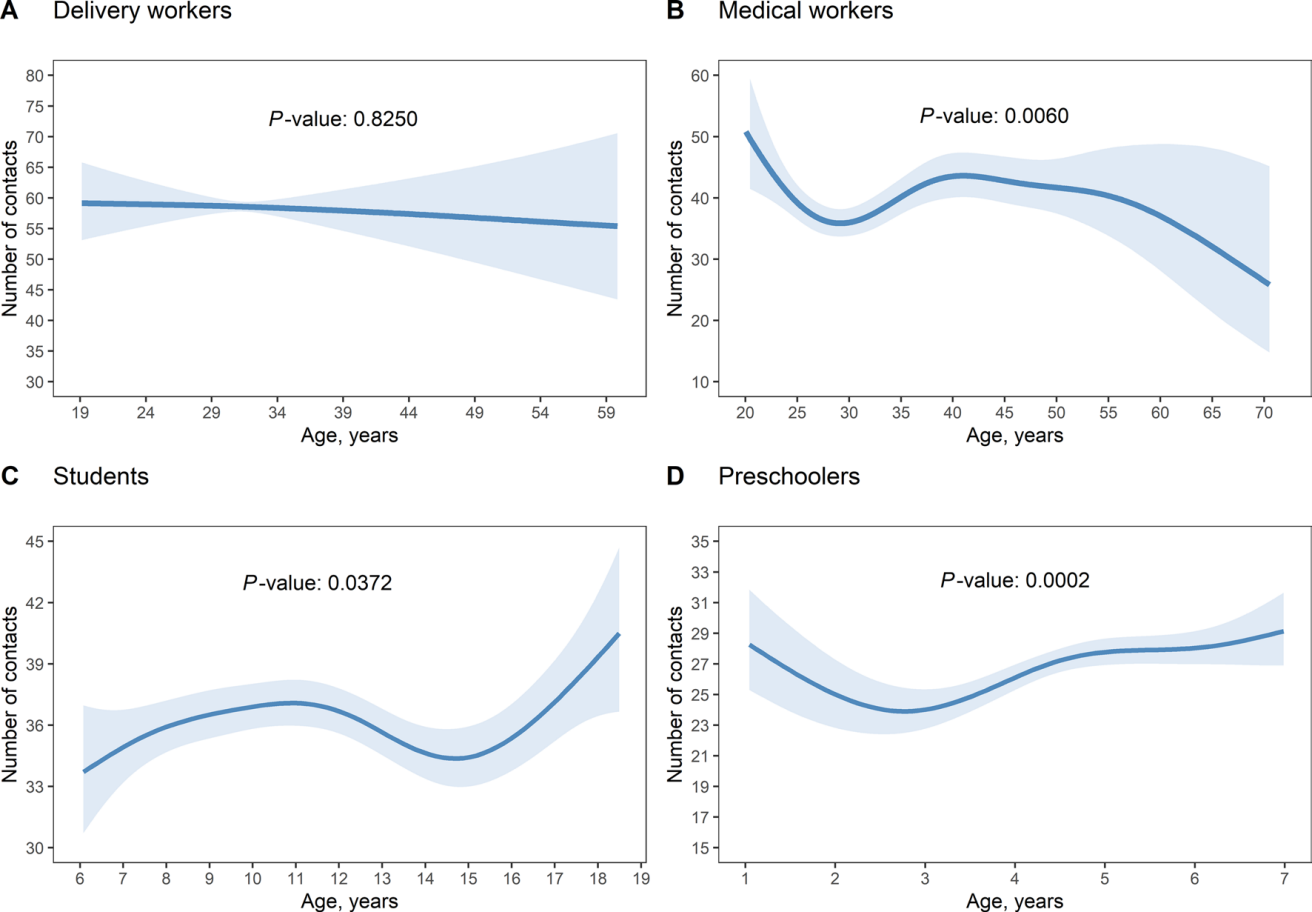
Estimated numbers of contacts in regression models for the different occupation groups, with 95% confidence intervals denoted by shaded regions

In the GAMM regression model (Table 2 and Fig. 5), all four occupation groups tended to show an increase in the contact number as the household size increased, although significant contributions were observed for only students and preschoolers. The number of contacts for female delivery workers was significantly lower than that for male delivery workers (*OR* = 0.50, 95% *CI*: 0.43–0.58). Household income per capita had a significant contribution to the number of contacts for preschoolers (*P*_50,000–99,999_ = 0.033) and students (*P*_10,000–29,999_ = 0.017). Students whose household incomes per capita per year exceeded CNY 100,000 had higher numbers of contacts than those whose household incomes were less than CNY 10,000 (*OR* = 1.23, 95% *CI*: 1.13–1.33). Students (*OR* = 1.18, 95% *CI*: 1.1–1.26) and preschoolers (*OR* = 1.14, 95% *CI*: 1.06–1.22) contacted more individuals on weekdays compared to weekends. Delivery workers and medical workers tended to contact more individuals on weekends, but these differences did not reach the level of significance (delivery workers: *P* = 0.354; medical workers: *P* = 0.851). Delivery workers (*OR* = 1.33, 95% *CI*: 1.01–1.75) and medical workers (*OR* = 1.18, 95% *CI*: 1.04–1.34) in Shanghai had higher contact numbers than those in Qinghai. The number of contacts had a significant nonlinear association with participant age among medical workers, students, and preschoolers. Delivery workers tended to have fewer contacts as their age increased; medical workers aged 25–35 and > 60 years typically had smaller numbers of contacts; and the number of contacts for students and preschoolers tended to increase with age (Fig. 5).

**Table 2:** Generalized additive model regression coefficient

Characteristics		Delivery workers		Medical workers		Students		Preschoolers	
		*OR* (95% *CI*)	*P* value	*OR* (95% *CI*)	*P* value	*OR* (95% *CI*)	*P* value	*OR* (95% *CI*)	*P* value
	Intercept	58.42 (42.55–80.21)	< 0.001	40.16 (34.15–47.23)	< 0.001	36.15 (34.43–37.94)	< 0.001	26.97 (24.99–29.11)	< 0.001
Household size	1–2 (Ref)	1	–	1	–	1	–	1	–
	3–4	0.96 (0.83–1.11)	0.570	1.05 (0.95–1.17)	0.297	1.05 (1–1.1)	0.047	1.02 (0.96–1.08)	0.483
	5 +	1.15 (0.92–1.44)	0.220	1.06 (0.91–1.23)	0.442	1.03 (0.97–1.09)	0.398	1.09 (1.01–1.17)	0.024
Sex	Male (Ref)	1	–	1	–	1	–	1	–
	Female	0.5 (0.43–0.58)	< 0.001	1.09 (0.98–1.22)	0.110	0.97 (0.93–1.01)	0.187	1 (0.95–1.05)	0.959
Household income per capita, CNY	< 10,000 (Ref)	1	–	1	–	1	–	1	–
	10,000–29,999	1.09 (0.92–1.29)	0.332	0.98 (0.83–1.16)	0.832	1.07 (1.01–1.13)	0.017	0.99 (0.91–1.07)	0.773
	30,000–49,999	1.1 (0.93–1.29)	0.268	1.01 (0.85–1.2)	0.923	1.12 (1.06–1.19)	< 0.001	1.03 (0.94–1.12)	0.541
	50,000–99,999	0.94 (0.8–1.1)	0.428	1.08 (0.93–1.26)	0.303	1.29 (1.21–1.38)	< 0.001	1.09 (1.01–1.18)	0.033
	≥ 100,000	0.94 (0.73–1.2)	0.600	1.04 (0.88–1.23)	0.611	1.23 (1.13–1.33)	< 0.001	1.08 (0.99–1.17)	0.070
Weekday	Weekend (Ref)	1	–	1	–	1	–	1	–
	Workday	0.92 (0.77–1.1)	0.345	0.99 (0.89–1.1)	0.851	1.18 (1.1–1.26)	< 0.001	1.14 (1.06–1.22)	< 0.001
Region	Qinghai (Ref)	1	–	1	–	1	–	1	–
	Shanghai	1.33 (1.01–1.75)	0.040	1.18 (1.04–1.34)	0.010	0.73 (0.66–0.81)	< 0.001	0.67 (0.61–0.73)	< 0.001
	Zhejiang	1.3 (0.97–1.74)	0.075	1.13 (0.99–1.29)	0.081	0.97 (0.89–1.06)	0.480	0.94 (0.86–1.01)	0.098

*OR* odd ratio; *CI* confidence interval, *CNY* Chinese Yuan

### Proportion of access to public places and mask wearing

**Fig. 6:**
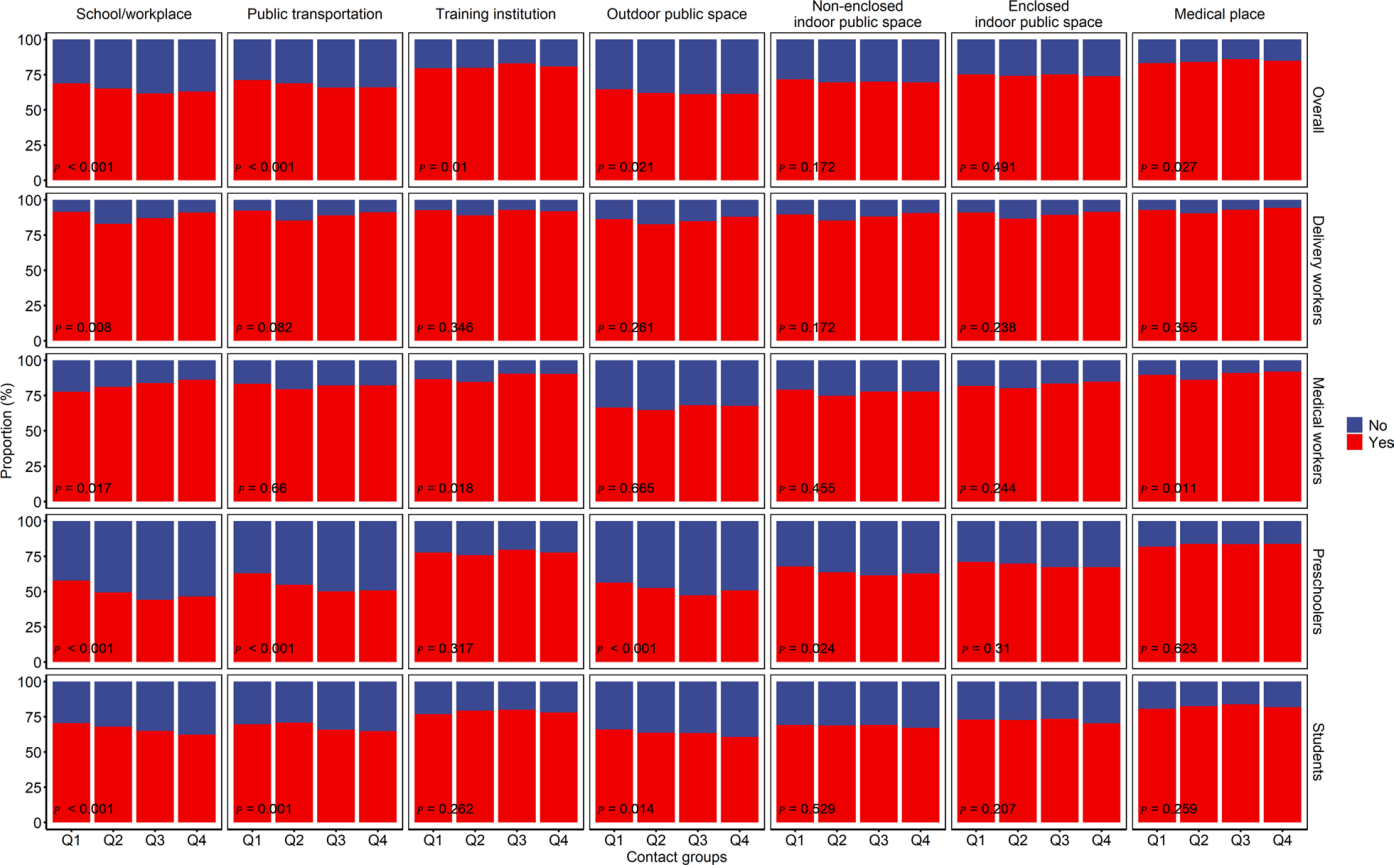
The proportion of participants who reported always wearing masks in different places, for different contact groups, stratified by occupation group

**Fig. 7:**
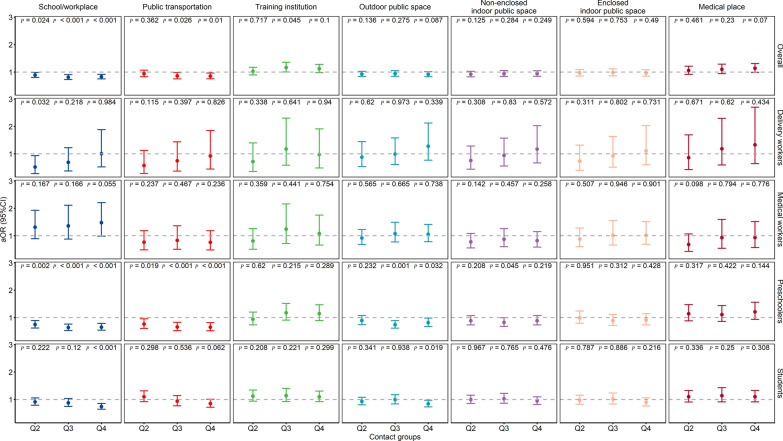
Associations of always wearing a mask with different contact levels. OR (dots) and 95% *CI* (error bars) were calculated from multivariate logistic regression after we adjusted for sex, province, and workday/weekday

All participants were asked to report on whether they went to seven listed places (school/workplace, public transportation, training institution, outdoor public space, non-enclosed indoor public space, enclosed indoor public space, and medical place) and how often they wore a mask in the visited places. Of the participants, 93.0% had gone to a school/workplace, while only 64.4% had gone to public transportation. The place most commonly visited by delivery workers and medical workers was a non-enclosed indoor public space, while the place most commonly visited by preschoolers and students was a school/workplace (Additional file 3: Table S2). Shanghai had the highest proportions of those who reported always wearing a mask for each of the seven places, with especially high proportions seen for training institutions and medical places. Females had higher proportions of always wearing masks in public places. Delivery workers had the highest proportion of always wearing masks, followed in decreasing order by medical workers, preschoolers, and students. On weekdays compared to weekends, participants reported higher proportions of always wearing masks in training institutions and medical places, but lower proportions in other places. The higher contact group had a lower proportion of always wearing masks (Additional file 1: Fig. S8). Our univariate logistic regression suggested that most of the above differences were significant (*P* < 0.05, Additional file 1: Fig. S9). After we adjusted for participants’ sex, province, and workday/weekday, the association between contact level and mask wearing differed across the four occupations (Figs. 6, 7). For delivery workers, it seemed that participants with a contact number in the middle of the range had a lower proportion of always wearing masks (*P*_School/workplace_ = 0.008). For medical workers, in workplaces (*P* = 0.017) and training institutions (*P* = 0.018), participants with more contacts had higher proportions of always wearing masks. For preschoolers and students, the proportion of respondents who reported always wearing masks in schools/workplaces (*P*_preschoolers_ < 0.001, *P*_students_ < 0.001) and public transportation (*P*_preschoolers_ < 0.001, *P*_students_ = 0.001) was lower for those with more contacts, whereas the proportion of respondents who reported always wearing masks in training institutions and medical places was higher for those with more contacts.

## Discussion

In this large-scale cross-sectional survey of self-reported contact patterns in representative high-risk groups from three provinces of China, we found that the average number of individual contacts per person per day is 3.14 (95% *CI*: 3.13–3.15). This is significantly lower than pre-pandemic numbers reported from Europe (13.4) [6], and Guangdong Province, China (16.7) [14], but similar to those reported in Canada (2.21–3.89) [16] and Europe (2–5) [17] after the outbreak of COVID-19. Our finding confirms that daily individual contacts were reduced several times during the existing COVID-19 prevention and control measures than pre-pandemic (14.6–18.8) [10], but higher than during the COVID-19 strict social-distancing period (2.0–2.3). Compared to slight fluctuation in daily individual contacts from periods of closure of schools and public places to the existing COVID-19 prevention and control measures, the numbers of group contacts had increased [10] and were significantly larger than the numbers of individual contacts across the four occupation groups. It meant that increases in contacts post-relaxation might be driven primarily by working or studying [17] during the COVID-19 social-distancing period [10].

Our contact matrix revealed that diagonal element strengths were obvious in all occupation groups and regions, and were particularly strong in the individual contacts. This indicates that all age groups were highly assortative, with interactions occurring much frequently with others of a similar age group. This characteristic is known to shape the transmission of infectious disease [18], suggesting that it is crucial to account for age-specific susceptibility to infection. For example, most individuals contacted by children and teenagers are of a very similar age [19–21], which is likely to be the main reason why children and teenagers represent an important conduit for the initial spread of close-contact infections in general and influenza in particular [1, 22]. Similar to the findings of Kiesha Prem’s research [23], we found that high assortativity of contacts is common in schools (here, students) and less apparent in working-age individuals in the workplace (here, medical workers and delivery workers). Medical workers and delivery workers need to contact patients and customers of all ages, and thus exhibited greater heterogeneity in the ages of their contacts. A more diverse age contact structure may provide a route for transmission to spread between medical workers (or delivery workers) and the rest of the population, resulting in a greater number of new infections [24].

Although the total contact number determines the potential frequency of exposure to infections, the risk of infection may depend more strongly on contact duration and physical contact [25, 26]. We considered a number of different measures for “closeness of contact,” including the duration and frequency of contact and whether skin-to-skin contact occurred. As previously reported [27, 28], these measures correlated highly with one another, such that longer-duration contacts tended to be frequent and involve physical contact (and vice versa). Importantly, more intimate contacts are likely to carry a greater risk of transmission [29]. Furthermore, these types of contact tend to occur in distinct social settings: skin-to-skin contacts typically occurred at home [30] or in the workplace, whereas non-physical contacts tended to occur in the transport sector. This variation has important implications for contact tracing during outbreaks of a new infection. Our results suggest that if efforts are concentrated on locating contacts in the home, school, and workplace, on average more than 75% of all contacts would be found.

Our research also found that the number of contacts increased with the household size, which is consistent with previous reports [10, 31]. Each year, a considerable sum is spent on private tutoring by Chinese families—in 2017 alone, an average household spent CNY 5616 (approximately USD 832) [32]. The reported contact numbers of students and preschoolers increased with the family income level, suggesting that students from higher-income families may have more opportunities to participate in various trainings and/or spend more time on social and leisure activities [31]. One study [33] showed that weekdays were associated with 23–28% more contacts than weekend days among students, perhaps indicating that students have fewer contacts with their classmates during weekends. Reported contact number for female delivery workers was significantly lower than that for male delivery workers, which might be due to better physical strength from male delivery workers. Our results showed that delivery workers (*OR* = 1.33, 95% *CI*: 1.01–1.75) and medical workers (*OR* = 1.18, 95% *CI*: 1.04–1.34) in Shanghai were more likely to have contact with others than their counterparts in Qinghai. This might reflect that contact rates and patterns among individuals is associated with population density [34]. Medical workers aged 25–35 and > 60 usually had lower numbers of contacts than aged 39–49, which might reflect that medical interns and doctors approaching retirement had fewer opportunities to contact patients.

Combined with social distancing, wearing a face mask can be effectively flatten the epidemic curve [35, 36]. Mask wearing was found to be significantly more prevalent among delivery workers and medical workers compared to students and preschoolers, suggesting that long-lasting COVID-19 behavior norms are likely to persist better in the occupational high-risk population than students and preschoolers. For preschoolers and students with more contacts, the proportion of those who reported always wearing masks was lower in schools/workplaces and public transportation than preschoolers and students with less contacts, suggesting that parents should make efforts to improve mask-wearing behavior in these groups [37]. Most notably, the percentages of mask wearing in the different contact groups did not differ much, regardless of whether or not the data were stratified by the occupation groups. Our findings suggest that participants with a lower contact number also maintained their mask-wearing behavior, suggesting that masks may have become a behavioral habit for Chinese residents [38]. Meanwhile, the higher contact group needs to be given more information of health education, and have more appropriate practices towards COVID-19, such as wearing masks. Previous research has found that sex, age, location, health consciousness, and knowledge of disease all influence whether members of the public wear a mask [39, 40]. We also found that females had higher proportions of always wearing masks in public places, because most Chinese residents, in particular women, are knowledgeable about COVID-19, hold optimistic attitudes, and have appropriate practices towards COVID-19 [41]. Men are known to engage more in risky active health behaviors because they are dangerous, thereby implying that they are strong and capable of performing dangerous activities [42]. Participants in Shanghai was observed to be more likely to keep wearing masks in public spaces, which might be due to the difference in education level, health awareness and estimated risk of COVID-19.

This study is prone to the limitations pertaining to social contact surveys. The contact survey and mask-wearing results are based on self-reported contacts, and may thus be affected by recall bias and self-reporting bias. In the future, this could be avoided by using a prospective design with advanced notification or instruction in face-to-face interviews. As this study focused on high-risk populations, caution should be used if seeking to apply our results to the general population.

## Conclusions

Contact tracing need to be concentrated at home, in school or workplace after an outbreak, as more than 75% of all contacts will be detected from these settings. Efforts should be made to improve the mask-wearing rate and age-specific health promotion measures aimed at reducing transmission for the younger demographic (especially the preschoolers and students), considering their susceptibility, lower mask-wearing rate and higher *q*-indices. Age-stratified and occupation-specific social contact research in high-risk groups can help inform policy-making decisions during the post-relaxation period of the COVID-19 pandemic.

## Supplementary Information

**Additional file 1. Figure S1.** The distributions of contacts’ age crossing different occupation. **Figure S2.** The proportion of reporting physical contacts in different contact durations stratified by province and occupation groups. **Figure S3.** The proportion of reporting physical contacts in different contact settings stratified by province and occupation groups. **Figure S4.** The proportion of reporting physical contacts in different contact relations stratified by province and occupation groups. **Figure S5.** The proportion of reporting physical contacts in different contact frequency stratified by province and occupation groups. **Figure S6.** Individual contact matrices of different province and occupations. **Figure S7.** Total contact matrices of different province and occupations. **Figure S8.** Proportion of always wearing masks in different places stratified by different characteristics. **Figure S9.** The association of always wearing masks and different characteristics of participants. **Figure S10.** The proportion of participants who reported always wearing masks in different places, for different contact groups, stratified by occupation group and province (A: Shanghai, B: Qinghai, C: Zhejiang).

**Additional file 2: Table S1.** Characteristics and number of contacts of participants stratified by provinces and occupations.

**Additional file 3: Table S2.** Proportion of participants had gone to the seven places (school/workplace, public transportation, training institution, outdoor public space, non-enclosed indoor public space, enclosed indoor public space and medical place) in the last month stratified by provinces and occupations.

## Data Availability

The data presented in this study are available on request from the corresponding author.
