# Supplementary material for: Patterns of human social contact and mask wearing in high-risk groups in China
Source: Infect Dis Poverty. 2022 Jun 18;11:69. doi: 10.1186/s40249-022-00988-8 (PMC9206088; doi:10.1186/s40249-022-00988-8)
Supplement: Supplementary file 1 — Additional file 1. Figure S1. The distributions of contacts’ age crossing different occupation. Figure S2. The proportion of reporting physical contacts in different contact durations stratified by province and occupation groups. Figure S3. The proportion of reporting physical contacts in different contact settings stratified by province and occupation groups. Figure S4. The proportion of reporting physical contacts in different contact relations stratified by province and occupation groups. Figure S5. The proportion of reporting physical contacts in different contact frequency stratified by province and occupation groups. Figure S6. Individual contact matrices of different province and occupations. Figure S7. Total contact matrices of different province and occupations. Figure S8. Proportion of always wearing masks in different places stratified by different characteristics. Figure S9. The association of always wearing masks and different characteristics of participants. Figure S10. The proportion of participants who reported always wearing masks in different places, for different contact groups, stratified by occupation group and province (A: Shanghai, B: Qinghai, C: Zhejiang). [file 40249_2022_988_MOESM1_ESM.docx]

**Supplemental Materials for**

**title**

Author

* Corresponding authors:

**This file includes:**

Table Sx to Table Sx

Figure Sx to Figure Sx

Figure S1
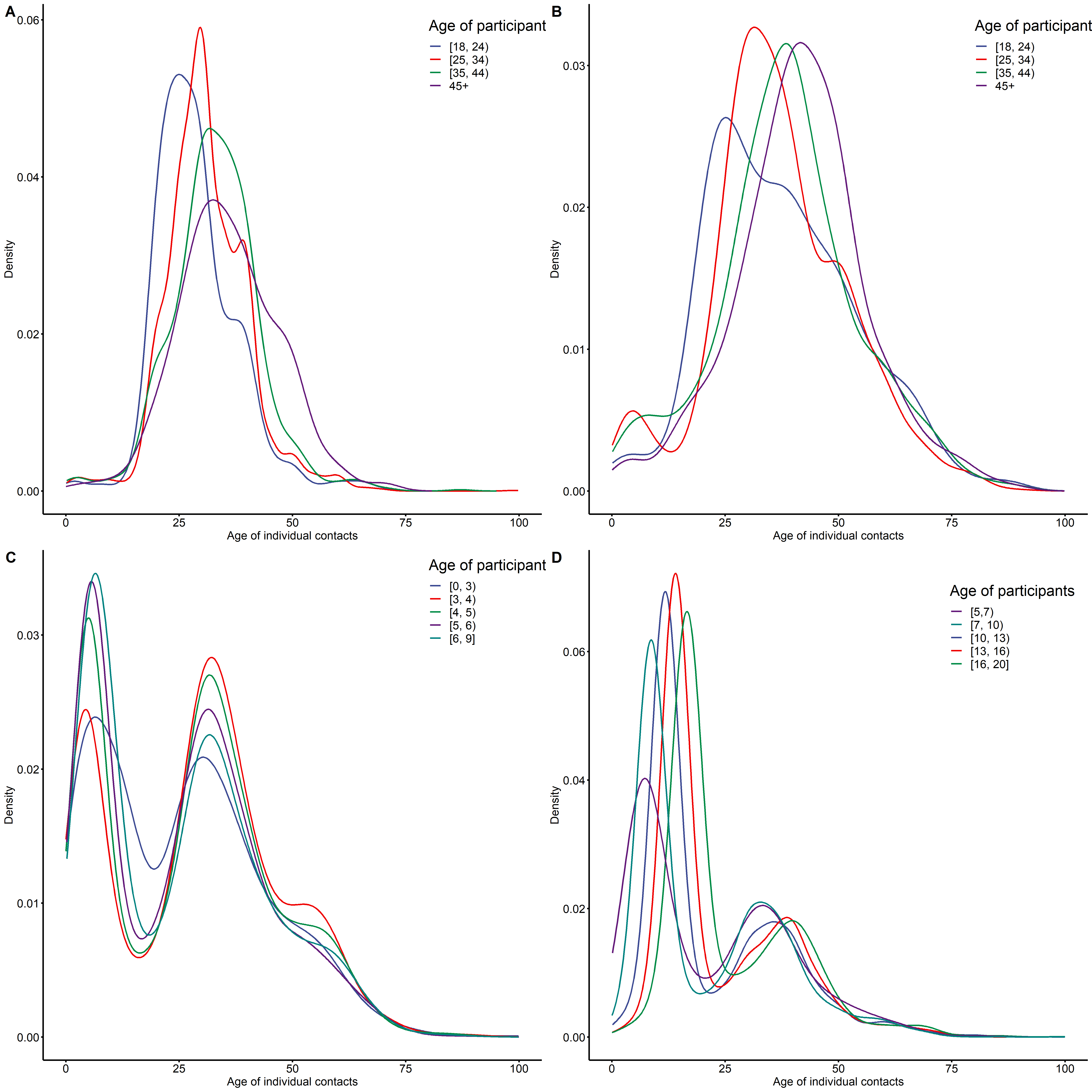
. The distributions of contacts’ age crossing different occupation (A: Deliverymen, B: medical workers, C: preschoolers, D: students) participants age groups. The distributions were fitted with Kernel density functions.


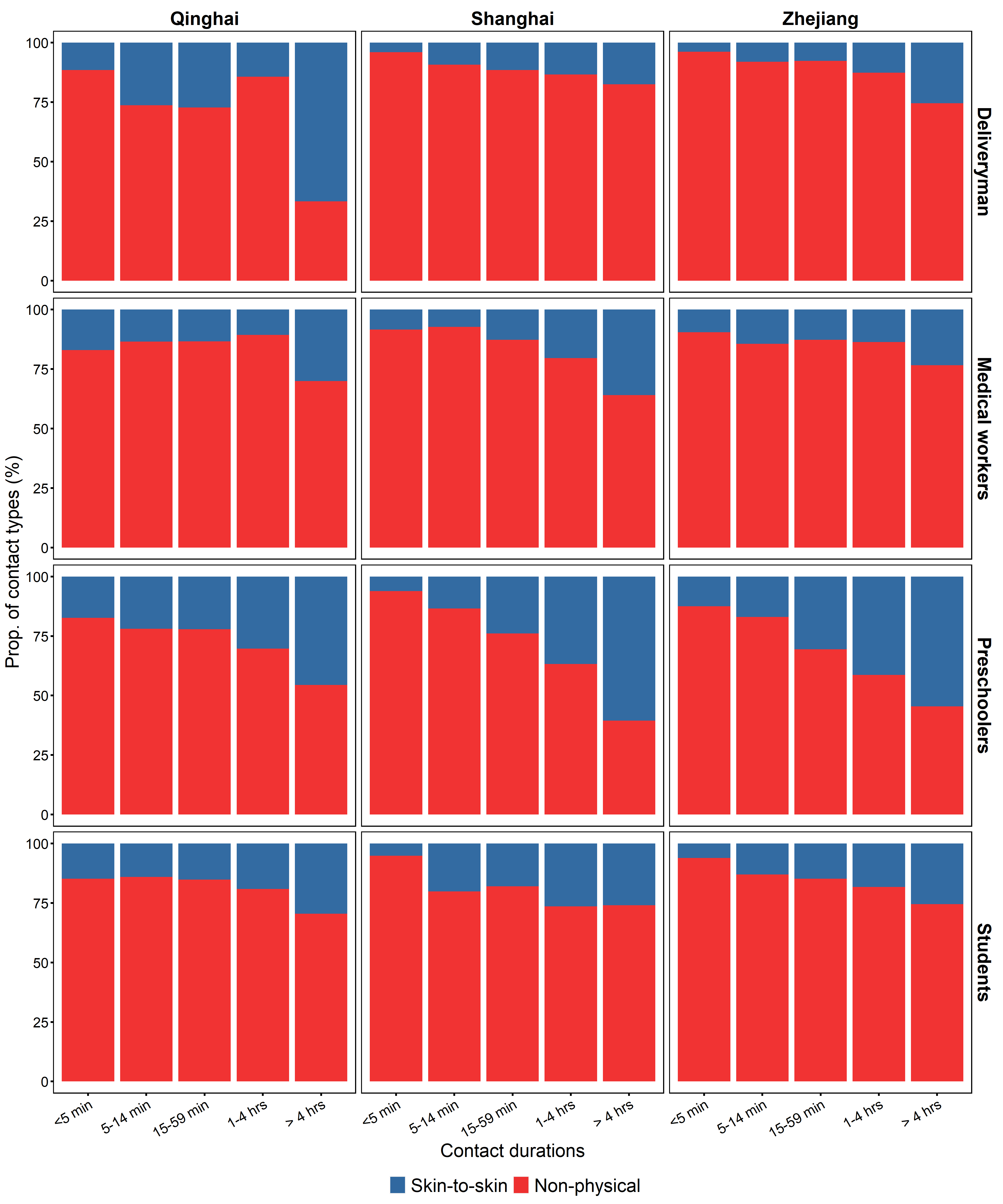
Figure S2. The proportion of reporting physical contacts in different contact durations stratified by province and occupation groups.


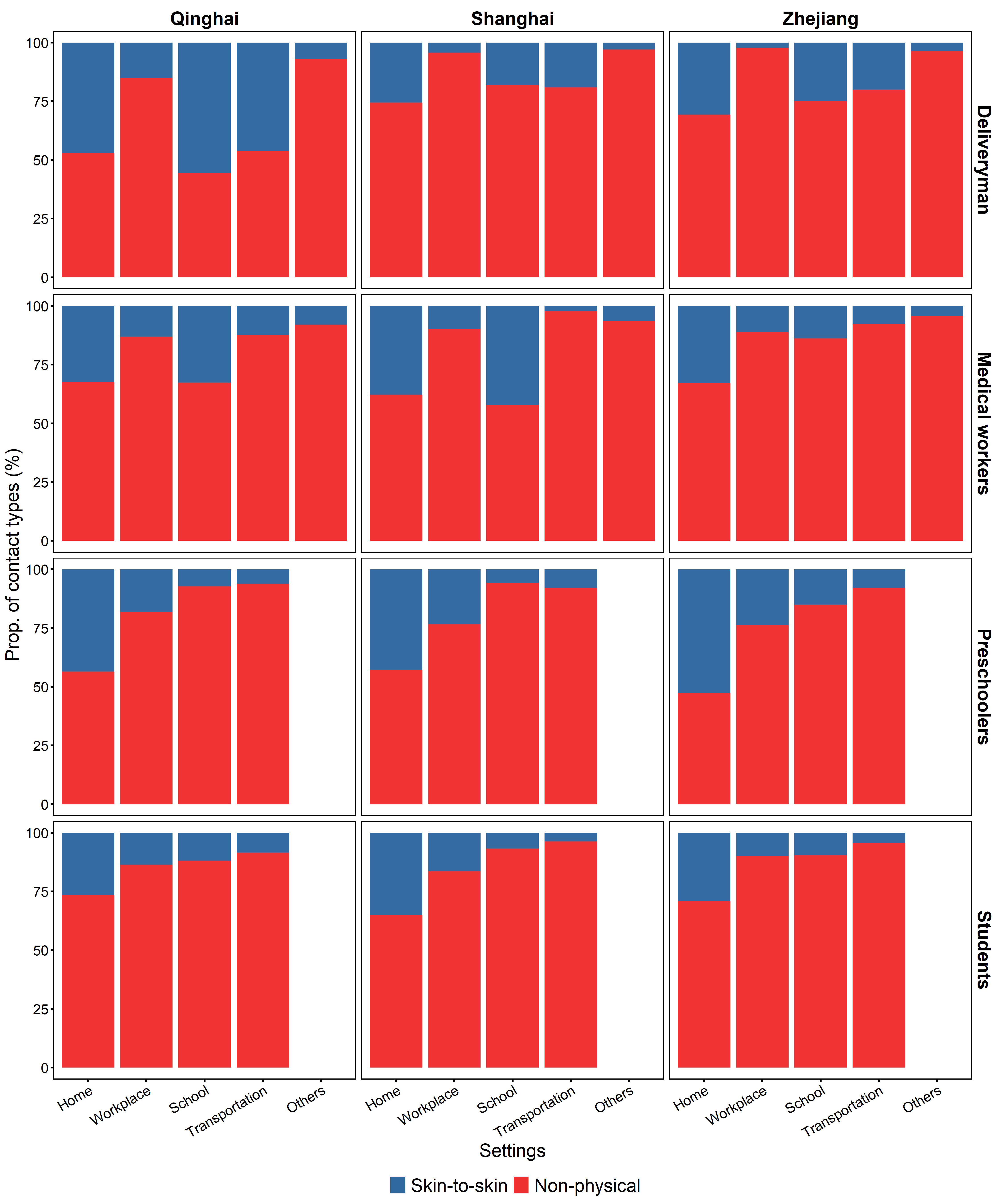
Figure S3. The proportion of reporting physical contacts in different contact settings stratified by province and occupation groups.


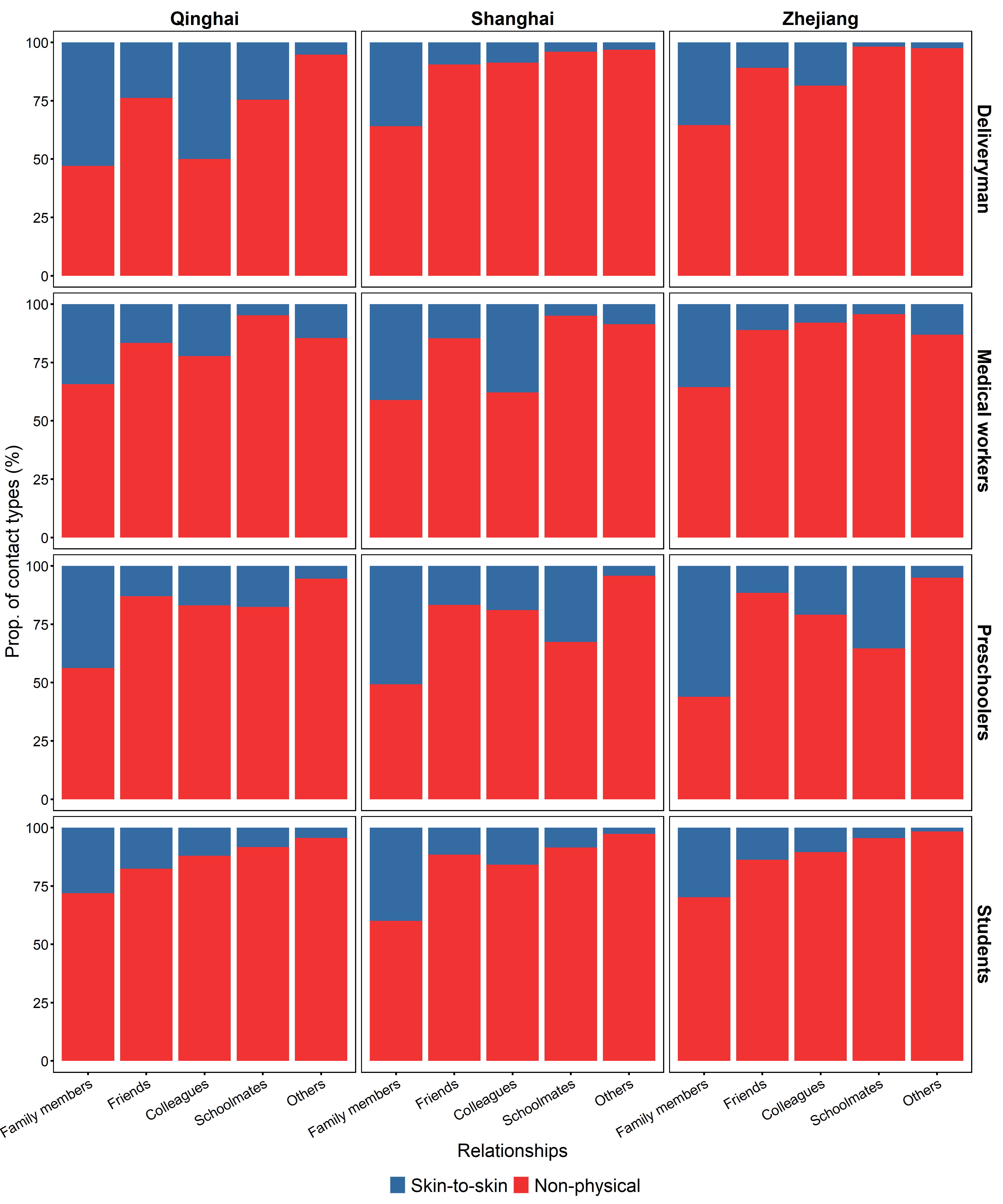
Figure S4. The proportion of reporting physical contacts in different contact relations stratified by province and occupation groups.


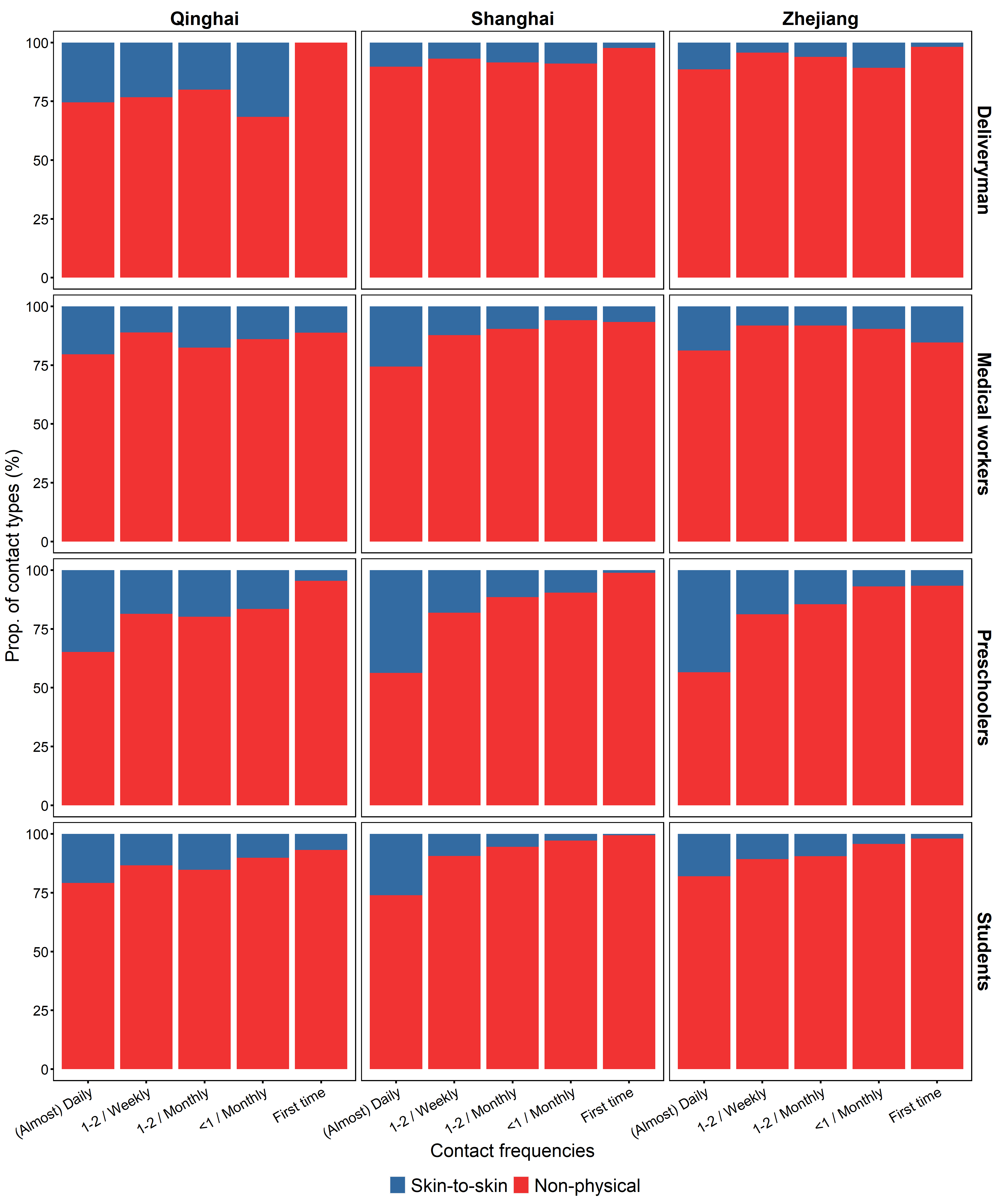
Figure S5. The proportion of reporting physical contacts in different contact frequency stratified by province and occupation groups.



Figure S6. Individual contact matrices of different province and occupations.



Figure S7. Total contact matrices of different province and occupations.


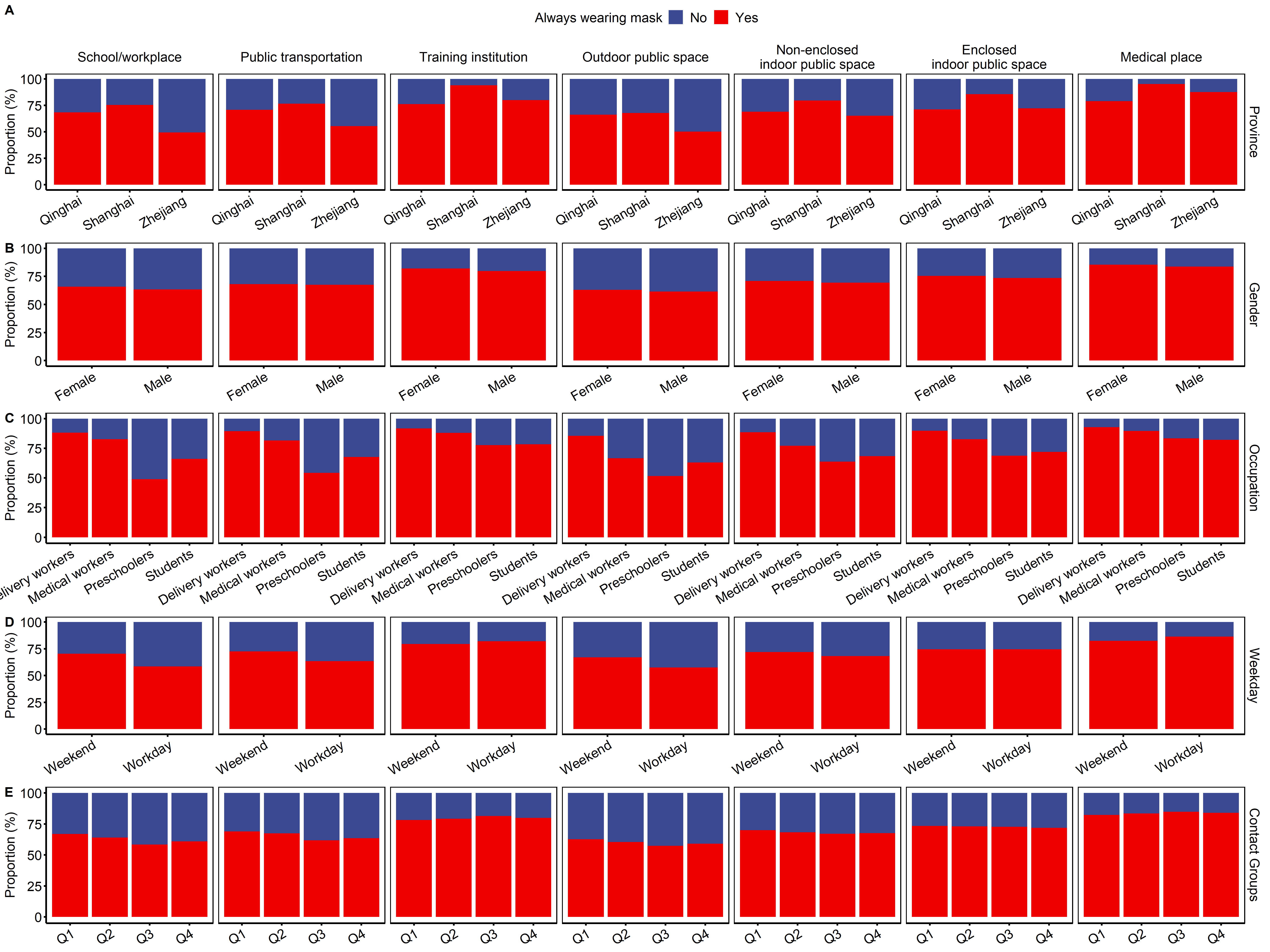
Figure S8. Proportion of always wearing masks in different places stratified by different characteristics.

Figure S9
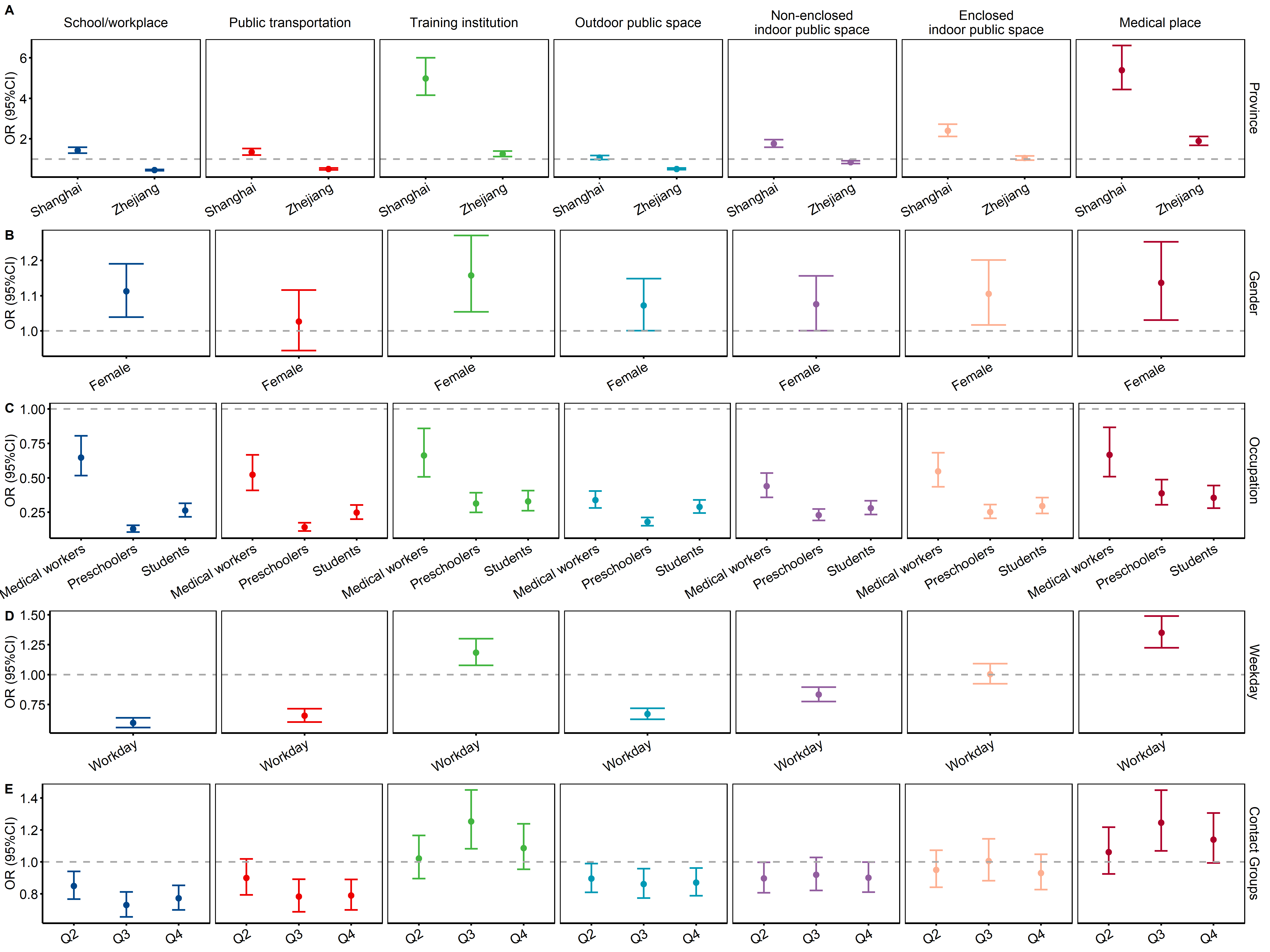
. The association of always wearing masks and different characteristics of participants. OR (dots) and 95%CI (error bars) was calculated from univariate logistics regression.


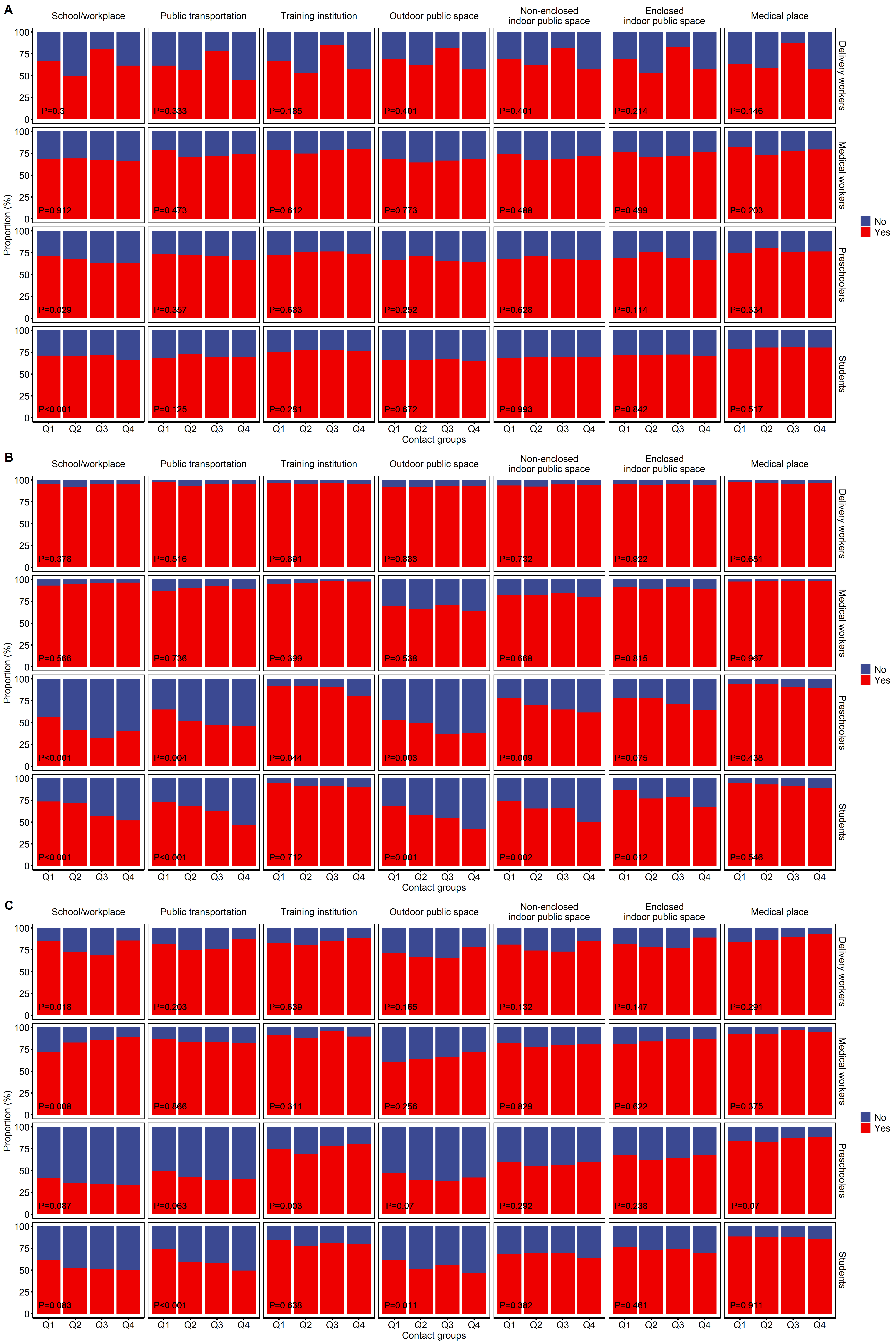


**Figure S10. The proportion of participants who reported always wearing masks in different places, for different contact groups, stratified by occupation group and province** (A: Shanghai, B: Qinghai, C: Zhejiang).
